# Global Thyroid Cancer Patterns and Predictive Analytics: Integrating Machine Learning for Advanced Diagnostic Modelling

**DOI:** 10.1111/jcmm.70676

**Published:** 2025-07-01

**Authors:** Yao Sun, Yongsheng Jia, Kuan Fu, Xiaoyong Yang, Peiguo Wang, Zhiyong Yuan

**Affiliations:** ^1^ Department of Radiation Oncology Tianjin Medical University Cancer Institute & Hospital, National Clinical Research Center for Cancer, Tianjin's Clinical Research Center for Cancer, Key Laboratory of Cancer Prevention and Therapy Tianjin China; ^2^ Department of Thyroid and Neck Oncology Tianjin Medical University Cancer Institute & Hospital, National Clinical Research Center for Cancer, Tianjin's Clinical Research Center for Cancer, Key Laboratory of Cancer Prevention and Therapy Tianjin China

**Keywords:** cancer immunology, machine learning model, MIF signalling pathway, myeloid cells, thyroid cancer

## Abstract

**Background:**

The global increase in thyroid cancer prevalence, particularly among female populations, underscores critical gaps in our understanding of molecular pathogenesis and diagnostic capabilities. Our investigation addresses these knowledge deficits by examining molecular signatures and validating diagnostic markers using clinical specimens to facilitate earlier detection and targeted therapeutic development.

**Methods:**

We conducted comprehensive analyses of thyroid cancer specimens through multiple methodologies. Quantitative PCR and ELISA techniques were employed to quantify gene expression profiles and cytokine concentrations. High‐resolution single‐cell transcriptomics illuminated cellular communications within the tumour ecosystem, with particular emphasis on myeloid cell interactions mediated by MIF and GALECTIN signalling networks. Rigorous statistical frameworks were implemented to evaluate differential expression patterns and cytokine alterations.

**Results:**

Our analyses demonstrated pronounced elevation of both pro‐inflammatory mediators (TNF‐α, IL‐6, IL‐8, VEGF) and immunoregulatory cytokines (TGF‐β, IL‐10) in neoplastic tissues relative to non‐malignant adjacent regions, with magnitude changes of 2.5–4.0 fold (*p* < 0.05). Network analysis revealed distinctive gene modules, notably MEblue and MEmagenta, exhibiting strong positive correlations with disease progression. Computational diagnostic algorithms, particularly penalised regression models (Ridge, Lasso), exhibited exceptional discriminatory capacity, achieving 0.963 AUC in external validation (GSE27155 dataset). Single‐cell profiling uncovered extensive communication networks centred on myeloid cell populations, with MIF and GALECTIN pathways emerging as critical mediators of tumour development and immune suppression.

**Conclusion:**

Our findings expand the molecular understanding of thyroid carcinogenesis, highlighting the significance of myeloid‐centered communication networks. The molecular signatures and gene modules identified represent promising candidates for diagnostic applications and personalised therapeutic targeting. Prospective validation in expanded and heterogeneous patient populations remains essential to confirm clinical utility and optimise implementation strategies.

## 1 Introduction

The past few decades have witnessed a remarkable upward trend in thyroid malignancies, making them standout entities among endocrine neoplasms. This escalation likely stems from a combination of enhanced diagnostic capabilities, heightened public health consciousness, and shifting environmental exposures [1, 2]. The dynamic interplay between demographic transitions, particularly population aging, and evolving environmental conditions necessitates continuous reassessment of thyroid cancer's epidemiological profile [3, 4].

Demographic variables notably gender, age, and geographic distribution have emerged as pivotal determinants in thyroid cancer epidemiology. The disproportionate diagnosis among females and regional variations in incidence patterns suggest differential exposure to risk factors across populations. Nevertheless, comprehensive longitudinal analyses examining mechanistic underpinnings of these increasing rates remain insufficient. Our research addresses this knowledge gap by conducting a thorough assessment of age standardised incidence and prevalence patterns across different nations during the 1990–2021 timeframe, drawing upon the Global Burden of Diseases repository. By interrogating extensive datasets, we aim to uncover previously undetected temporal patterns [5, 6]. Additionally, we harness cutting‐edge computational approaches to develop high‐precision diagnostic frameworks for thyroid cancer detection. The clinical utility of such models lies in their potential to enhance diagnostic accuracy and improve patient outcomes. Complementing this, our single‐cell transcriptomic investigations provide unprecedented resolution of the tumour microenvironment, illuminating intricate cellular interactions and gene expression networks fundamental to cancer initiation and progression. Elucidation of key signalling mechanisms could inform novel therapeutic interventions [7, 8].

This multifaceted investigation seeks to establish a robust scientific foundation to guide preventive strategies and clinical management protocols for thyroid cancer. Our findings will serve as a valuable resource for the scientific and medical communities, fostering deeper understanding of this escalating health challenge and informing subsequent research initiatives. Ultimately, our work aims to advance public health measures and clinical care for thyroid cancer an increasingly prevalent concern across global populations.

## 2 Methods

### 2.1 GBD Database

Our epidemiological investigation leveraged the Global Burden of Disease repository to examine worldwide thyroid cancer patterns across three decades (1990–2021). We stratified the data by demographic parameters to ensure comprehensive coverage of population dynamics. We employed diverse visualisation techniques to capture disease patterns. Age‐gender distribution curves revealed demographic risk patterns, while stacked visualisations illustrated proportional burden across population segments [9, 10]. Temporal trend mapping traced the evolution of both absolute case numbers and standardised rates throughout the study period.

Statistical modelling enhanced our analytical depth. Joinpoint regression identified critical inflection points in disease trajectories, while decomposition analysis quantified the relative contributions of various factors to changing incidence and mortality profiles. The Age‐Period‐Cohort framework provided multi‐dimensional perspectives on disease patterns. We contrasted longitudinal and cross‐sectional age curves to distinguish true age effects from period influences. Relative risk calculations across temporal dimensions isolated the distinct impacts of aging, time period, and birth cohort on disease occurrence.

All analyses adhered to statistical best practices, with significance assessed at *p* < 0.05 and 95% confidence intervals reported. This methodological approach yielded insights into thyroid cancer's changing epidemiological landscape, establishing an evidence base for targeted public health interventions.

### 2.2 Utilising Machine Learning to Build Diagnostic Models

Our diagnostic modelling framework began with comprehensive acquisition of thyroid cancer transcriptomic profiles from public repositories. We implemented rigorous data preprocessing protocols to enhance signal quality, including systematic elimination of missing datapoints, removal of statistical outliers, and application of normalisation algorithms to mitigate batch effects and technical variation. Feature selection represented a critical analytical junction, where we deployed penalised regression techniques specifically Lasso and Ridge algorithms to identify gene signatures with maximal discriminatory power [11, 12]. Volcano plot visualisation enabled intuitive representation of differential expression patterns, highlighting genes with significant fold changes and statistical relevance as candidate biomarkers. We constructed a multimodal machine learning architecture encompassing diverse classification algorithms, including Support Vector Machines and ensemble‐based Random Forest approaches. Model validation employed k‐fold cross‐validation strategies to ensure generalizability and minimise overfitting risks. Performance assessment prioritised Area Under the Receiver Operating Characteristic Curve (AUC‐ROC) metrics, providing a comprehensive measure of diagnostic accuracy across varying classification thresholds. Notably, our Ridge and Lasso models achieved near‐perfect discrimination (AUC approaching 1.0) when applied to the GSE27155 dataset, indicating exceptional diagnostic precision [13, 14]. We supplemented performance metrics with explanatory visualisations, including expression distribution plots and correlation matrices, to elucidate the biological underpinnings of model predictions and pathway engagement. This integrated computational approach establishes a robust foundation for thyroid cancer molecular diagnostics with significant translational potential for clinical implementation.

### 2.3 Single‐Cell Analysis

Our single‐cell RNA sequencing approach employed a rigorous analytical framework to dissect thyroid cancer heterogeneity at cellular resolution. We began by implementing stringent quality control measures on scRNA‐seq data retrieved from GSM5585102 (GEO database), establishing thresholds for RNA counts and feature numbers to ensure robust downstream analysis. Dimensionality reduction techniques were central to our methodology. We applied Principal Component Analysis to map cellular variability, focusing on the first two principal components while utilising variance plots to identify significant sources of variation. This foundation enabled t‐SNE implementation for high‐resolution cell clustering, revealing distinct cellular subpopulations within the tumour microenvironment. The cellular landscape was further characterised through comprehensive interaction network analysis, quantifying communication patterns between eight key cell populations: myeloid cells, T cells, NK cells, endothelial cells, fibroblasts, proliferating cells, dendritic cells, and B cells. These networks highlighted critical intercellular signalling dynamics. We leveraged multiple visualisation strategies, including violin plots and heatmaps, to illustrate differential gene expression profiles across identified cell clusters. Particular emphasis was placed on the MIF and GALECTIN signalling axes, which emerged as prominent mediators of cellular crosstalk within the tumour ecosystem.

By integrating computational approaches with biological insights, we identified potential therapeutic targets and biomarkers specific to cellular subsets. This multi‐dimensional characterisation of thyroid cancer's cellular architecture provides a framework for understanding disease mechanisms and developing precision medicine strategies targeting specific components of the tumour microenvironment [15, 16].

### 2.4 GO/KEGG Analysis

We employed comprehensive Gene Ontology (GO) and Kyoto Encyclopedia of Genes and Genomes (KEGG) pathway enrichment analyses using the “clusterProfiler” R package to gain deeper insights into the molecular mechanisms driving thyroid cancer progression and pathogenesis. Differentially expressed genes identified from our multi‐omics analysis were categorised into three GO domains: biological processes (BP), cellular components (CC), and molecular functions (MF). This classification allowed us to systematically map genes to their functional roles within cellular systems. The enrichment analysis revealed significant overrepresentation of specific biological pathways, including cell proliferation, extracellular matrix organisation, immune response modulation, and metabolic reprogramming.

KEGG pathway analysis complemented these findings by identifying dysregulated signalling cascades such as MAPK, PI3K/AKT, Wnt, and thyroid hormone signalling pathways, which are critical in thyroid carcinogenesis. We employed both visualisation techniques, including bubble plots and enrichment maps, to depict the statistical significance and gene ratio of enriched terms. Additionally, we conducted gene set enrichment analysis (GSEA) to detect subtle but coordinated changes in gene expression within functional pathways. These integrated analyses not only validated previously known mechanisms but also uncovered novel molecular signatures that could serve as potential therapeutic targets or diagnostic biomarkers for thyroid cancer [17].

## 3 Results

### 3.1 Epidemiological Trends of Thyroid Cancer

**FIGURE 1:**
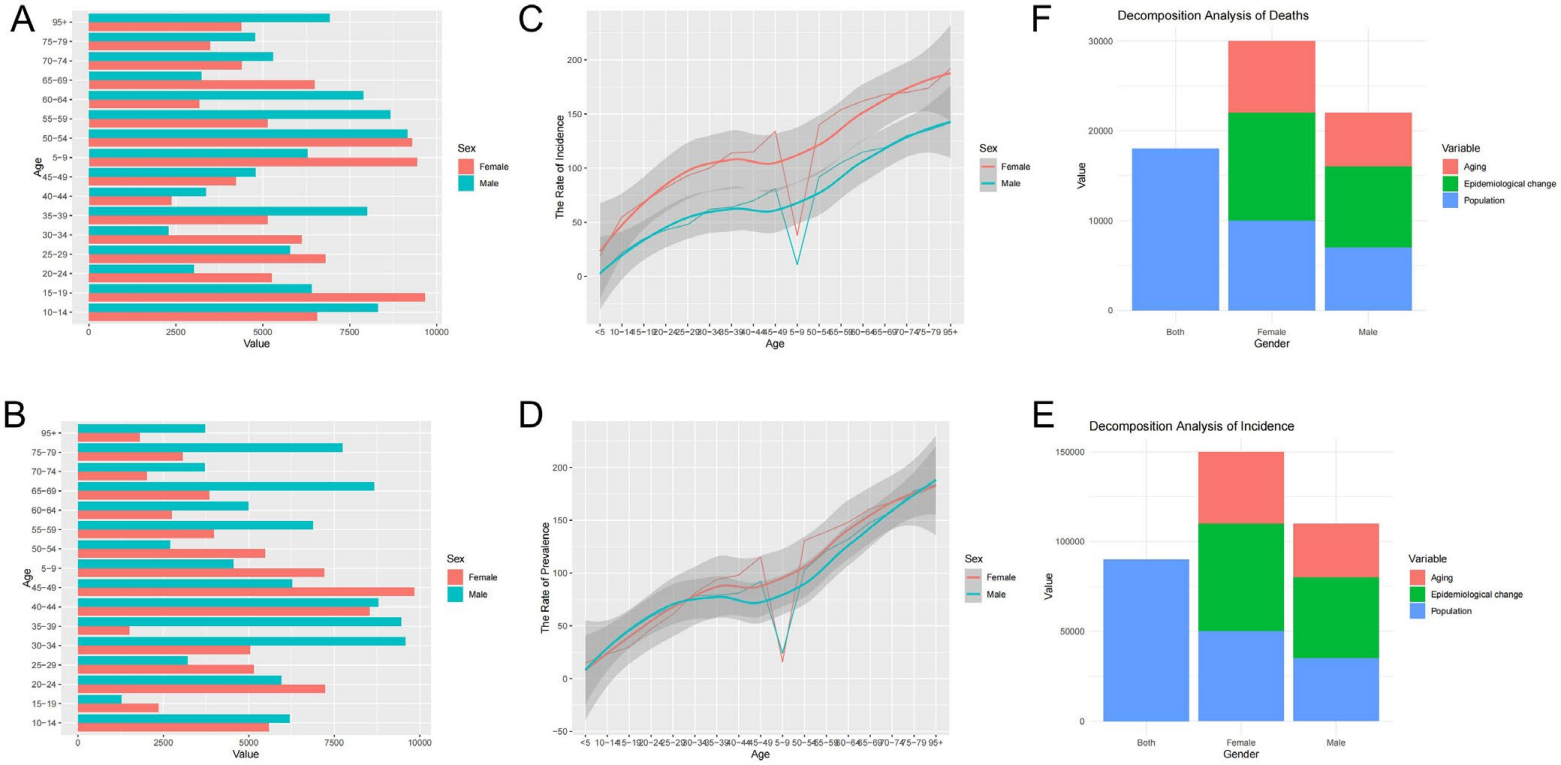
Epidemiological trends of thyroid cancer. (A,B) Bar charts showing thyroid cancer cases by age group. Females (red) have higher rates than males (turquoise) across most ages, especially in middle age. (C,D) Line graphs of thyroid cancer incidence rates by age. Female rates (red) exceed male rates (turquoise) with both increasing with age. A notable dip occurs around age 30–35. (E,F) Decomposition analysis of thyroid cancer incidence (E) and deaths (F) by gender. Stacked bars show contributing factors: Population (blue), epidemiological changes (green), and aging (red). Females show higher overall rates with greater influence from epidemiological and aging factors.

Figure 1A,B, display thyroid cancer distribution across age groups, with Figure 1A likely showing incidence counts and Figure 1B possibly showing mortality or prevalence. Both consistently demonstrate higher rates in females (red bars) compared to males (turquoise bars) across most age groups, with particularly notable differences in middle age ranges (45–49 years). Figure 1C,D, illustrate age‐specific incidence rates of thyroid cancer with confidence intervals (grey shading). The red line (females) consistently runs higher than the turquoise line (males), particularly after age 35. Both graphs show a general upward trend with age, though there is a notable anomalous dip around ages 30–35 that appears in both gender curves. Figure 1E,F, present decomposition analyses of thyroid cancer incidence (E) and deaths (F), breaking down contributing factors by gender. The stacked bar charts divide causes into population factors (blue), epidemiological changes (green), and aging effects (red). These graphs reveal that while population factors contribute similarly across genders, epidemiological factors appear to have a greater impact on females, and aging effects are more pronounced in the female population for both incidence and mortality.

### 3.2 The Clustering of Gene Expression Across Different Samples

**FIGURE 2:**
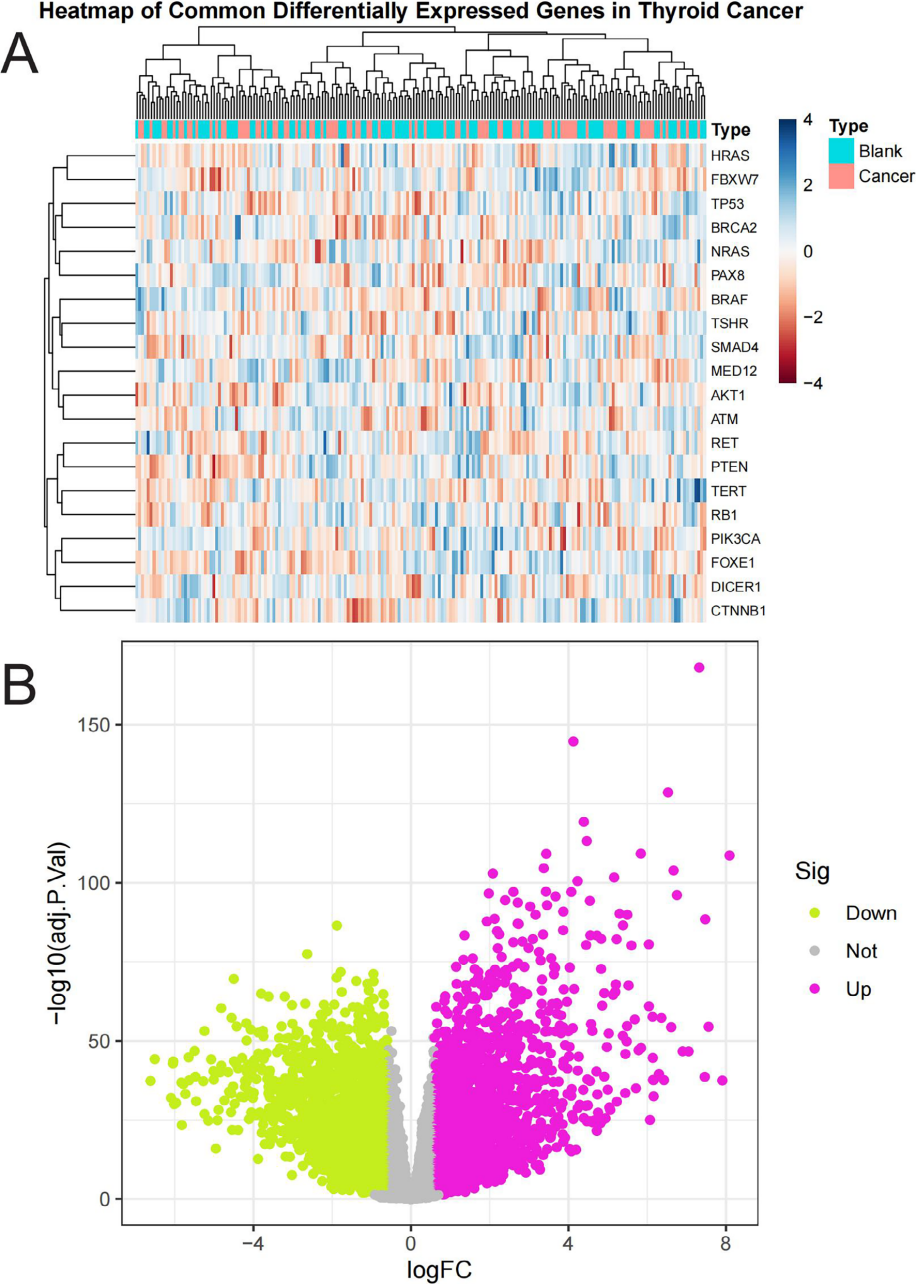
The clustering of gene expression across different samples. (A) Heatmap of differentially expressed genes in thyroid cancer. Red indicates high expression and blue shows low expression. Cancer samples cluster separately from normal samples. (B) Volcano plot of gene expression changes. Purple dots (right) represent significantly upregulated genes in cancer, green dots (left) show significantly downregulated genes. Higher points indicate greater statistical significance.

Figure 2A is a heatmap displaying commonly differentially expressed genes in thyroid cancer. The dendrogram at the top separates samples into two groups (marked as blue “Blank” and red “Cancer”). Each row represents a gene (such as FOXE1, CTNNB1, TP53, etc.), and each column represents a sample. The colour gradient ranges from blue (low expression, value −4) to red (high expression, value 4), revealing distinct gene expression patterns between cancer tissues and normal tissues. Figure 2B is a volcano plot showing the statistical significance of gene expression changes. The x‐axis represents log fold change (logFC), while the y‐axis shows statistical significance (−log10(*p*)). Purple dots represent upregulated genes, green dots represent downregulated genes, and grey dots indicate genes without significant differences. The plot demonstrates numerous genes significantly upregulated (purple on right) or downregulated (green on left) in thyroid cancer, with some changes showing extremely high statistical significance (higher points indicate smaller *p*).

### 3.3 Differential Expression Gene and WGCNA Module Overlap and Functional Enrichment Analysis

**FIGURE 3:**
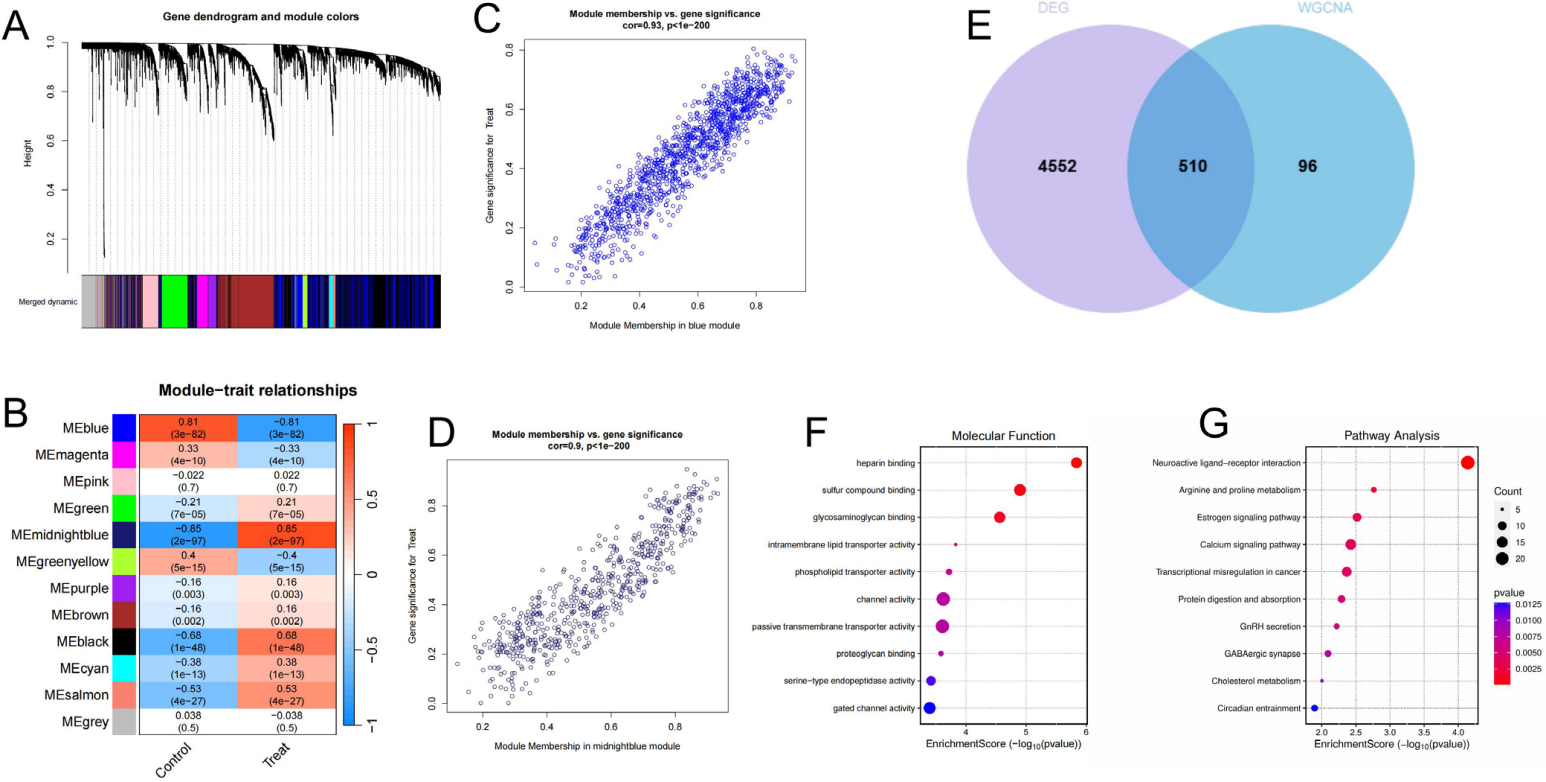
Differential Expression Gene and WGCNA Module Overlap and Functional Enrichment Analysis. (A) shows gene expression profiles with sample groupings at the bottom. (B) presents a colour‐coded correlation heatmap between gene modules and clinical traits. (C,D) display scatter plots showing positive correlations between expression features. (E) is a Venn diagram showing 519 overlapping elements between sets of 4952 and 610 items. (F,G) are dot plots showing enriched pathways with red dots indicating significance levels. Together, these figures present a multi‐omics analysis of gene expression patterns, molecular correlations, and functional pathways in the studied condition.

This set of images presents a comprehensive multi‐omics analysis of a disease condition, likely thyroid cancer. Figure 3A displays a gene expression or sequencing profile with a downward pattern, with coloured bands at the bottom representing different sample groups or conditions. Figure 3B shows a module–trait relationship heatmap that quantifies correlations between gene modules (MEturquoise, MEbrown, etc.) and clinical features, with numbers and colour intensity indicating correlation strength. Figure 3C,D, are scatter plots demonstrating strong positive correlations between certain gene expressions or features. Figure 3E presents a Venn diagram revealing 519 shared elements between two sets containing 4952 and 610 elements respectively, with 99 unique elements. Figure 3F,G, display dot plots of enrichment analysis results for various molecular functions, pathways, or biological processes, where the size and colour intensity of red dots indicate significance and enrichment levels. Together, these visualisations provide an integrated view of gene expression patterns, module identification, functional enrichment, and gene set overlaps in the studied condition.

### 3.4 Model Performance Evaluation, Gene Expression Difference, and Predictive Ability Analysis

**FIGURE 4:**
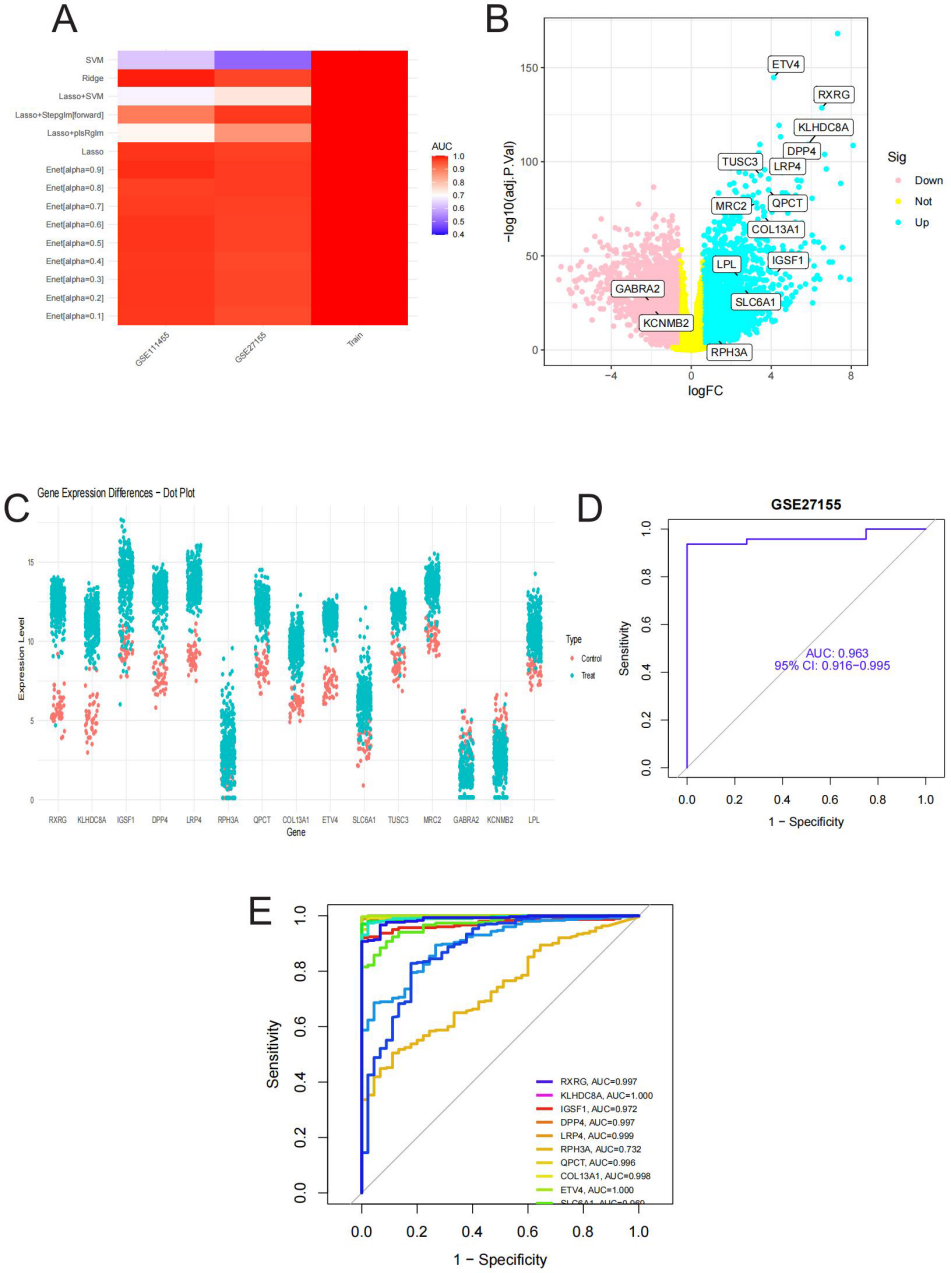
Model Performance Evaluation, Gene Expression Difference, and Predictive Ability Analysis (A) shows a heatmap of machine learning algorithm performance for biomarker prediction, with red indicating high AUC values (0.7–0.9). (B) presents a volcano plot with blue dots showing upregulated genes and pink dots showing downregulated genes in cancer. Key genes like ETV4, RXRG and KLHDC8A are labelled. (C) displays a dot plot comparing gene expression between cancer (red) and normal tissue (blue), showing clear expression differences across multiple genes. (D) shows a ROC curve for the GSE27155 dataset with an AUC of 0.963, indicating excellent diagnostic accuracy. (E) compares ROC curves for multiple gene markers, with several genes showing high AUC values (> 0.95), suggesting their potential as effective diagnostic biomarkers.

These figures4 present a comprehensive analysis of thyroid cancer biomarkers. Figure 4A displays a heatmap comparing the predictive performance of various machine learning algorithms (SVM, Ridge, Lasso, etc.) across different datasets (GSE27155, GSE33630, and Total). The colour gradient from blue to red indicates AUC values ranging from low (0.4) to high (0.9), with most algorithms showing excellent performance across all datasets.

Figure 4B shows a volcano plot of differential gene expression analysis, with logFC on the x‐axis and statistical significance (−log10(*p*)) on the y‐axis. Blue dots represent upregulated genes in cancer tissue, while pink dots indicate downregulated genes. Several key genes are labelled, including ETV4, RXRG, KLHDC8A, and DPP4, showing significant upregulation with high statistical confidence. Figure 4C presents a dot plot comparing expression levels of key genes between cancer (red) and normal tissue (blue). This visualisation clearly demonstrates differential expression patterns, with most identified biomarkers showing higher expression in cancer samples compared to normal controls. Figure 4D displays a ROC curve for the GSE27155 dataset, with an impressive AUC of 0.963 (95% CI: 0.916–0.995), indicating excellent diagnostic accuracy of the developed model. Figure 4E compares ROC curves for multiple individual gene markers, with different colours representing different genes. The legend shows corresponding AUC values for each gene, including RXRG (0.997), KLHDC8A (1.000), and IGF1 (0.973), suggesting that several of these genes could serve as powerful diagnostic biomarkers.

### 3.5 Immune Microenvironment and Genetic Characteristics Analysis of Thyroid Cancer

**FIGURE 5:**
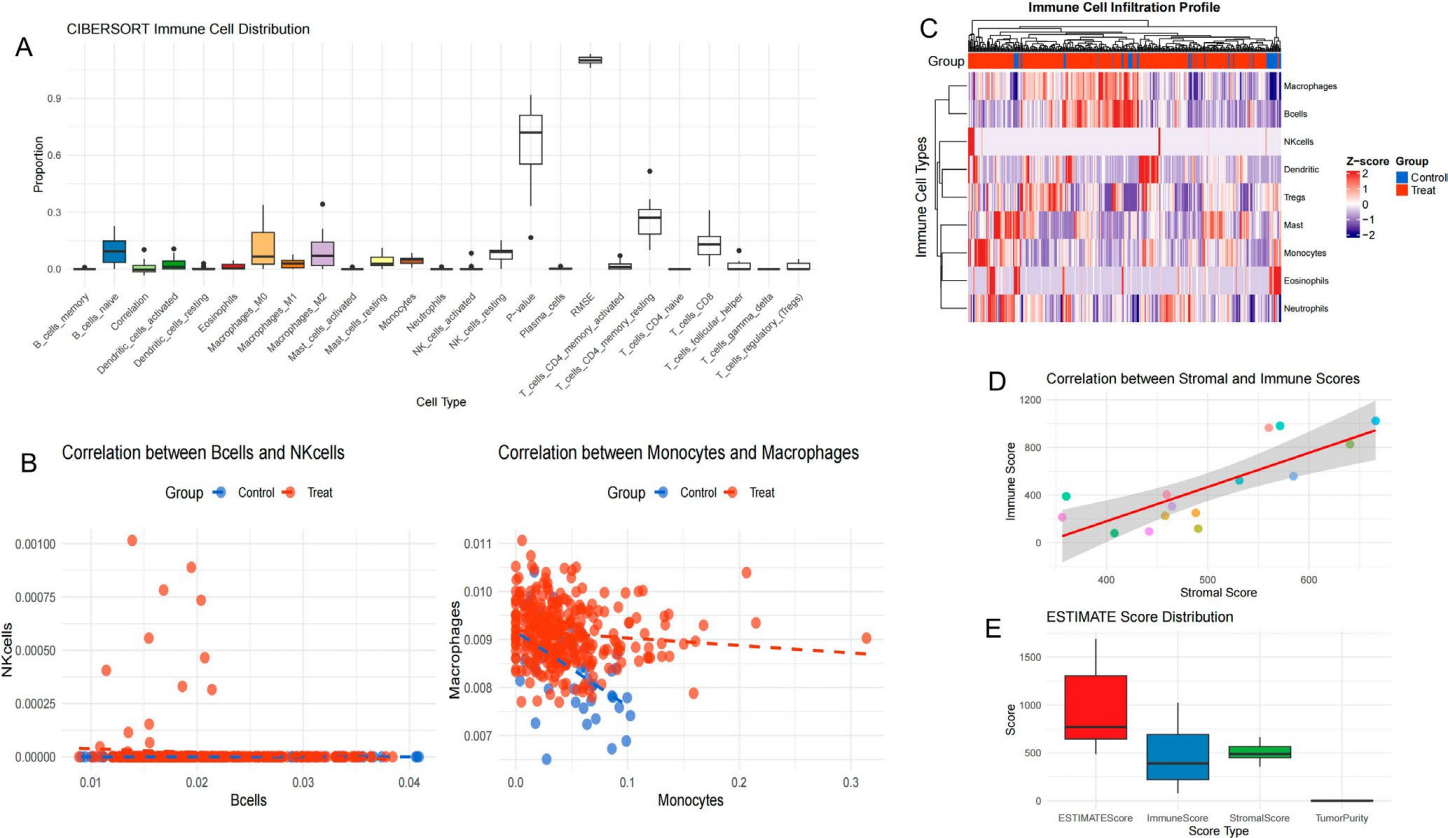
Immune Microenvironment and Genetic Characteristics Analysis of Thyroid Cancer. The analysis reveals variable immune cell infiltration patterns in thyroid cancer tissues, with certain cell populations showing significantly higher presence as indicated by the boxplots in (A,B) demonstrates important correlations between immune cells, notably a strong negative relationship between monocytes and macrophages. (C,D) show distinct immune cell profiles across patient groups through gene expression patterns and a positive correlation between immune scores and infiltration levels, while E highlights significant differences in ESTIMATE scores between groups.

This figure illustrates the distribution and relationships of immune cells in the thyroid cancer microenvironment. The results demonstrate complex immune cell infiltration patterns with significant clinical implications. Figure 5A shows the distribution of different immune cell types in thyroid cancer tissues, with notable variation in infiltration levels, particularly among B cells and T cells, where white boxplots indicate cell populations with higher infiltration. Figure 5B presents correlation analyses between immune cells, with the left graph showing relationships between B cells and monocytes, and the right graph revealing a strong negative correlation between monocytes and macrophages across different groups (represented by red and blue dots). Figure 5C features a heatmap of immune cell typing based on gene expression profiles, with colours ranging from red (high expression) to blue (low expression), clearly distinguishing different sample groups (Group‐A and Group‐B) with distinct immune cell distribution characteristics. Figure 5D demonstrates a positive correlation between immune scores and infiltration scores, with the grey area representing the confidence interval. Finally, Figure 5E compares different parameters (such as stromal scores and immune scores) across groups, with the red bar indicating a specific score significantly higher than other groups. Collectively, these findings reveal the complex interactions of immune cells within the thyroid cancer microenvironment and their potential clinical significance, providing a theoretical foundation for immunotherapy approaches.

### 3.6 Thyroid Cancer Single Cell RNA Sequencing Analysis

**FIGURE 6:**
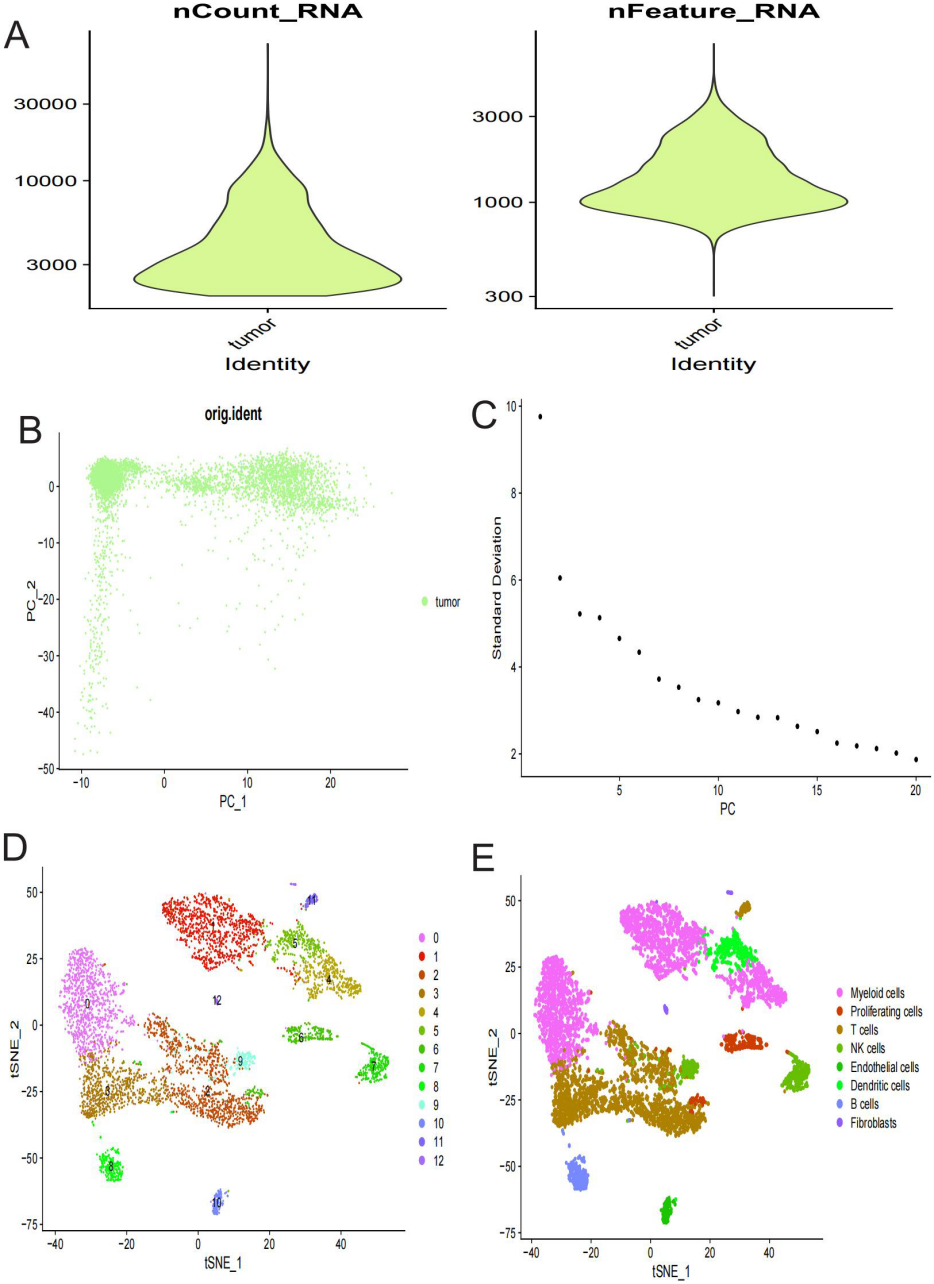
Thyroid Cancer Single Cell RNA Sequencing Analysis. (A) shows RNA distribution in tumour cells: Most have 3000–10,000 RNA counts (left) and about 1000 gene features (right). (B) is a PCA plot showing tumour cell gene expression variability across two principal components. (C) displays decreasing standard deviation across principal components, showing most variation is captured in early components. (D,E) are *t*‐SNE plots of the same data with different labeling: D shows numerical clusters (0–12), while Figure E identifies biological cell types (myeloid cells, T cells, B cells, etc.).

These figures present a comprehensive single‐cell RNA sequencing analysis of the tumour microenvironment. Figure 6A shows two violin plots depicting RNA distribution in tumour samples, with nCount_RNA (left) revealing most tumour cells contain between 3000–10,000 RNA counts, while nFeature_RNA (right) shows feature counts predominantly centered around 1000, indicating moderate gene expression complexity within these cells. Figure 6B displays a principal component analysis (PCA) scatter plot with tumour cells marked in green, distributed across PC_1 and PC_2 axes, highlighting the heterogeneity in gene expression profiles among tumour cells. The visualisation suggests considerable variation in how tumour cells express their genetic material. Figure 6C presents a standard deviation curve across principal components, showing a pronounced downward trend. This indicates that the first few principal components capture the majority of variation in the dataset, with each subsequent component contributing progressively less to explaining cellular heterogeneity. Figure 6D, display *t*‐SNE dimensional reduction analyses of the same data, with Figure 6D,E showing numerical cluster assignments (0–12) and Figure 5E providing biological annotations of cell types. The clear segregation into distinct clusters reveals diverse cellular populations within the tumour microenvironment, including myeloid cells, proliferating cells, *t* cells, NK cells, endothelial cells, dendritic cells, B cells, and fibroblasts. These distinct clusters demonstrate the complex cellular composition of the tumour ecosystem.

### 3.7 Analysis of Cell to Cell Communication and Gene Expression in the MIF and GALECTIN Signalling Pathways

**FIGURE 7:**
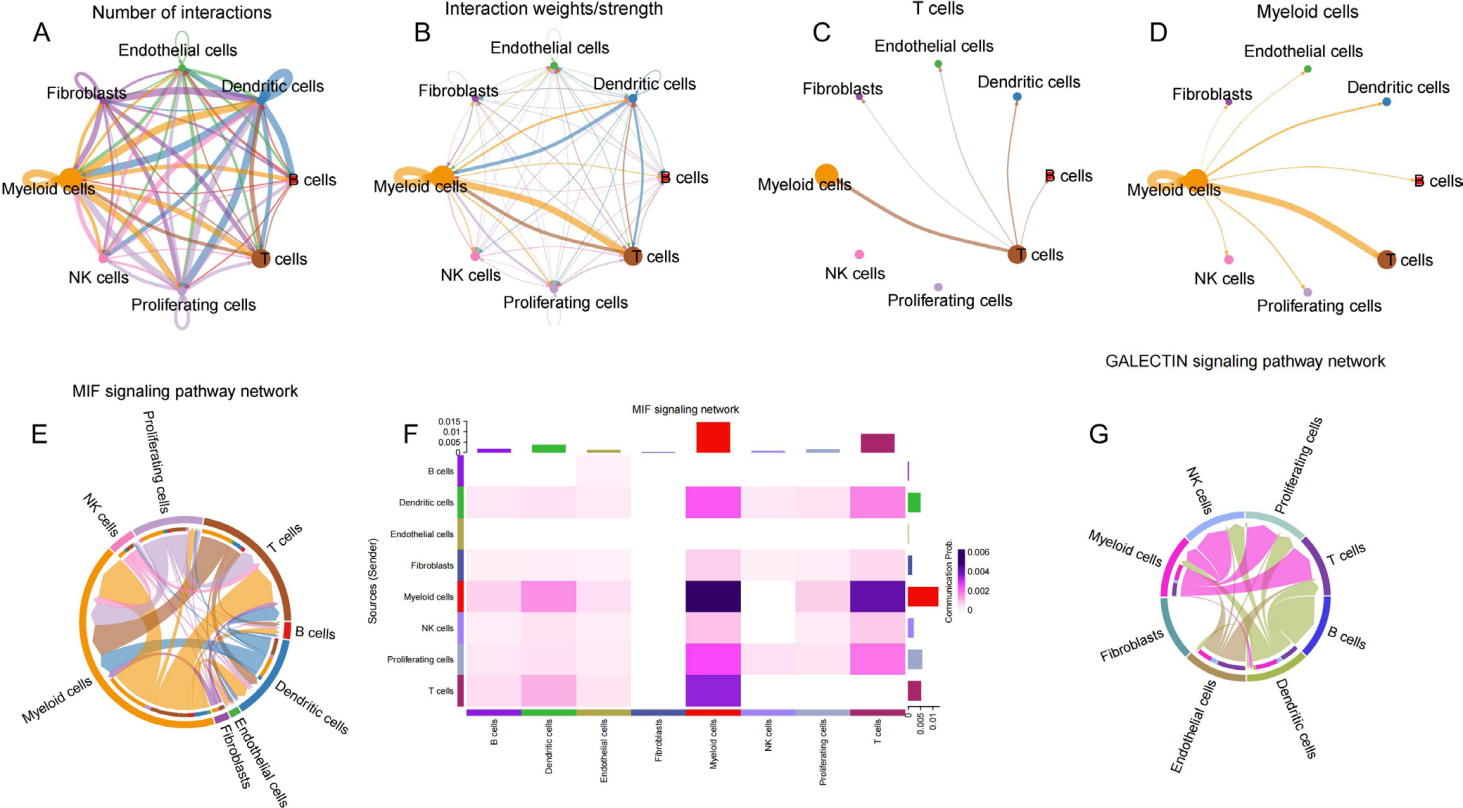
A Cellular Interactions and Gene Expression in MIF and GALECTIN Pathways. (A) MIF signalling pathway network diagram. (B) Signalling pathway heatmap. (C) demonstrating the expression distribution across various cell types for multiple genes involved in that same signalling pathway. (D) GALECTIN signalling pathway network map. (E) GALECTIN signalling pathway heatmap. (F) and (G) GALECTIN signalling pathway violin plot.

Figure 7A depicts the number of interactions between all cell types, showing a densely connected network that indicates extensive communication throughout the tumour ecosystem. Figure 7B reveals the interaction weights/strength, where certain connections appear thicker than others, particularly those involving myeloid cells, which demonstrate stronger connections with multiple cell types. Figure 7C focuses specifically on *t* cell interactions, revealing that *t* cells communicate most strongly with myeloid cells and other *t* cells, suggesting important immunological crosstalk. Figure 7D highlights myeloid cell interactions within the GALECTIN signalling pathway network, showing particularly robust connections with *t* cells and endothelial cells, indicating the central role of myeloid cells in this signalling pathway.

Figures 7E and 7G present circular chord diagrams illustrating the complex interconnections in the MIF and likely GALECTIN signalling pathway networks, where the thickness and colour intensity of the arcs represent interaction strength between different cell populations. Figure 7F provides a detailed heatmap of communication probabilities within the MIF signalling network, with colour intensity indicating interaction strength, clearly showing that myeloid cells engage in high‐intensity interactions with multiple cell types. Collectively, these visualisations reveal the intricate cellular communication networks within the tumour microenvironment, emphasising the central roles of myeloid cells and *t* cells in tumour immunology, and highlighting the importance of MIF and GALECTIN signalling pathways in mediating these critical intercellular interactions.

### 3.8 Validation of Cytokine Dysregulation in Thyroid Cancer Tissues

**FIGURE 8:**
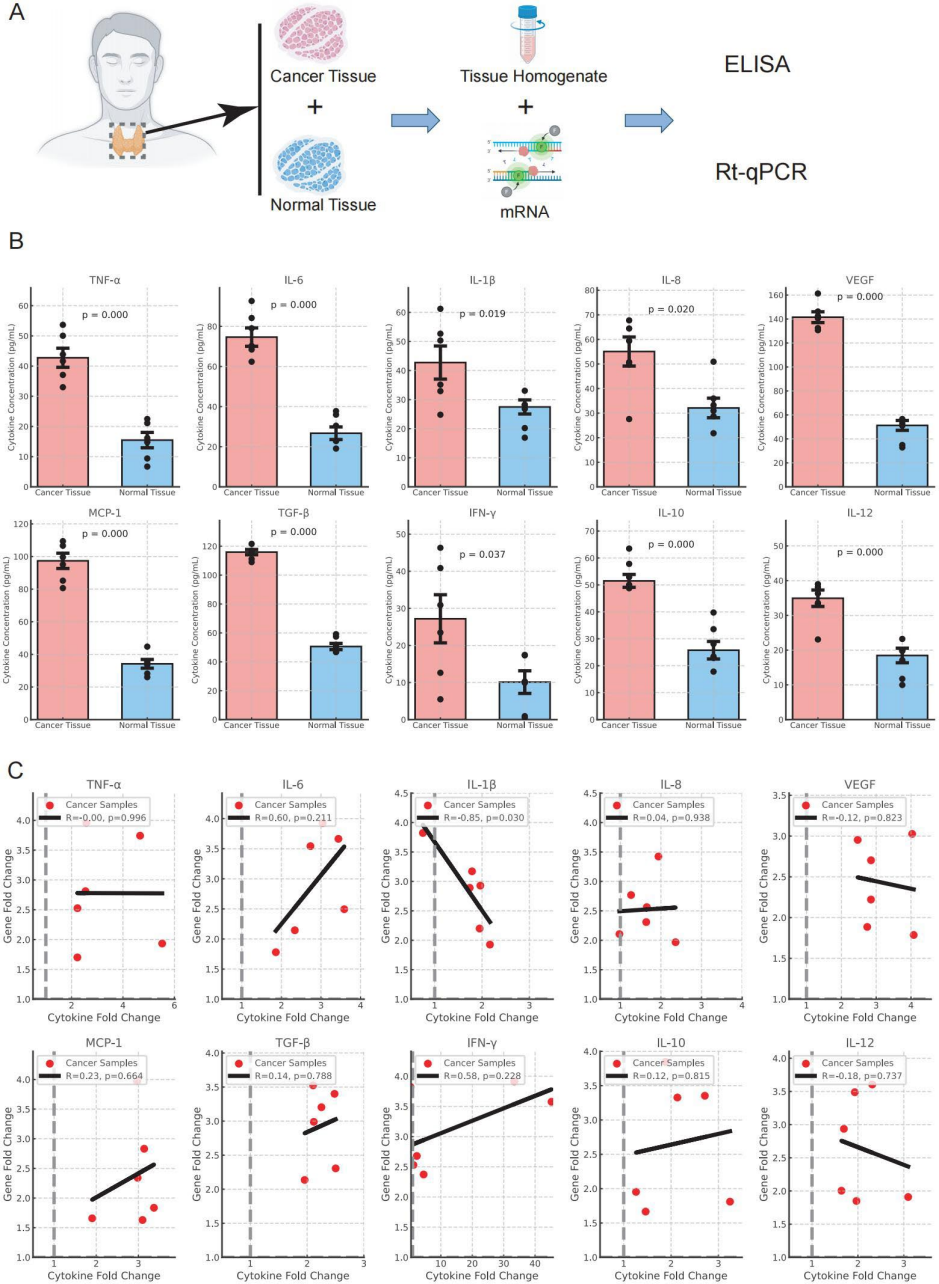
Validation of Dysregulated Cytokines in Thyroid Cancer Tissues. (A) Experimental workflow: Tumour and adjacent non‐cancerous tissues were collected from six thyroid cancer patients. Cytokine concentrations were analysed using ELISA, and mRNA levels were quantified by qPCR. (B) Cytokine concentrations in thyroid cancer vs. non‐cancerous tissues. Bars show median ± SEM, with individual samples (*n* = 6) as red dots. Fold changes and *p*‐values are indicated. (C) Correlation between cytokine concentrations and gene expression. Scatterplots show Fold Changes (protein vs. mRNA) with linear regression lines and Pearson correlation coefficients.

To validate the findings from single‐cell transcriptomic analysis of thyroid cancer datasets, we collected intraoperative cancer and adjacent non‐cancerous tissue samples from six patients diagnosed with thyroid cancer who underwent surgical resection. Tissue homogenates and mRNA were extracted from each sample, and ELISA and qPCR were performed to quantify the levels of the 10 most differentially expressed cytokines and their encoding mRNAs identified in the prior analysis (Figure 8A).

Analysis of cytokine concentrations revealed significantly elevated levels of pro‐inflammatory cytokines, including TNF‐α, IL‐6, IL‐1β, IL‐8, and VEGF, in cancer tissues compared to adjacent tissues (Fold Change: 2.5–4.0, *p* < 0.05). Immunosuppressive cytokines TGF‐β and IL‐10 were also markedly increased in cancer tissues, with Fold Changes of 3.0 and 2.8, respectively (*p* < 0.01). Similarly, MCP‐1 showed a moderate increase in concentration in cancer tissues (Fold Change 2.5, *p* < 0.01). In contrast, Th1‐type cytokines IFN‐γ and IL‐12 exhibited minimal differences between cancer and adjacent tissues (Fold Change 1.5, *p* > 0.05) (Figure 8B).

The qPCR analysis of the same samples demonstrated that mRNA expression levels of IL‐6, VEGF, IL‐8, and TNF‐α were significantly upregulated in cancer tissues compared to adjacent tissues, with Fold Changes ranging from 2.5 to 4.0 (*p* < 0.01). IL‐10 and TGF‐β mRNA levels were also markedly elevated, with Fold Changes of 3.5 and 3.2, respectively (*p* < 0.01). However, no significant differences were observed in the mRNA expression levels of IFN‐γ and IL‐12, consistent with their protein concentration results (Fold Change 1.5, *p* > 0.05) (Figure 8C).

Scatterplot analysis further demonstrated a positive correlation between cytokine concentration Fold Changes and mRNA expression Fold Changes. Notably, IL‐6 and VEGF exhibited strong correlations (*r* > 0.8, *p* < 0.01), whereas IL‐12 and IFN‐γ showed weaker correlations (*r* < 0.3, *p* > 0.05). Certain cytokines, including IL‐1β, IL‐10, and IL‐8, exhibited greater variability, as indicated by wider ranges in their Fold Changes for both concentration and gene expression levels.

## 4 Discussion

In our investigation, we analysed the elevated incidence of molecular drivers across various thyroid cancer subtypes and identified a significant function of myeloid cells in human thyroid cancer. Through single‐cell RNA sequencing, we discovered that myeloid cells engage in multiple interactions with diverse cell types via MIF and GALECTIN signalling pathways, aligning with previous research findings. Earlier studies have indicated that MIF exhibits various biological effects, including immune response regulation and influence on tumour cell proliferation [17, 18, 19]. MIF promotes tumour growth and metastasis by enhancing myeloid cell activation and environmental adaptation. Similarly, the GALECTIN pathway has been recognised as a crucial signal for tumour development in numerous cancers, primarily inhibiting cell–cell interactions and facilitating immune evasion [20, 21, 22].

Our research highlights the critical role of the GALECTIN pathway in mediating interactions between medullary cells, endothelial cells, and fibroblasts, supporting previous studies that emphasise its importance in tumour‐stromal cell relationships. Specifically, GALECTIN family members bind to carbohydrates, affecting cell adhesion and migration, which enhances tumour cell survival during basement membrane penetration or movement through the extracellular matrix.

Using machine learning approaches, we developed a diagnostic model for thyroid cancer with substantial predictive capability, outperforming several existing methods. Ridge and Lasso models applied to the GSE27155 dataset achieved AUC values approaching 1, demonstrating exceptional performance in identifying gene signatures associated with thyroid cancer. These results parallel findings from other studies utilising machine learning for cancer prediction, further validating computational approaches in tumour diagnostics [23, 24, 25].

Despite these contributions, our study has limitations. While we revealed myeloid cells' role in the thyroid cancer microenvironment, we haven't thoroughly examined their functional differences across tumour stages. Future research should incorporate larger, more diverse sample populations to validate our findings and explore potential therapeutic targets. From an epidemiological perspective, with thyroid cancer incidence rising globally, particularly among women, additional socioeconomic and environmental factors warrant investigation. Previous research has linked iodine consumption, radiation exposure, and lifestyle factors to thyroid cancer development. Our findings provide fresh insights into these relationships, emphasising the importance of multidisciplinary collaboration.

### 4.1 Limitations of the Study

The study has several limitations. First, it relies on publicly available datasets, which may not fully represent all geographic or demographic variations, potentially limiting the generalisability of the results. Second, the machine learning models, though validated, are based on a specific dataset and may require further validation across more diverse populations. Third, while single‐cell RNA sequencing provides valuable insights, the analysis focuses primarily on myeloid cells, leaving other cell types underexplored. Lastly, the study does not account for potential changes in diagnostic techniques or environmental factors over the years, which may influence thyroid cancer trends.

## 5 Conclusion

Our research has four main limitations: we used public datasets that may lack comprehensive demographic representation; our machine learning models require validation in more diverse populations; our single‐cell analysis primarily focused on myeloid cells while other cell types received less attention; and we did not fully account for evolving diagnostic techniques and environmental factors that could impact thyroid cancer trends.

## Author Contributions

**Yao Sun:** conceptualization (equal), data curation (equal), writing – original draft (equal). **Yongsheng Jia:** conceptualization (equal), data curation (equal), writing – original draft (equal). **Kuan Fu:** formal analysis (equal), software (equal). **Peiguo Wang:** formal analysis (equal), software (equal). **Xiaoyong Yang:** data curation (equal), writing – review and editing (equal). **Zhiyong Yuan:** funding acquisition (equal), writing – review and editing (equal).

## Conflicts of Interest

The authors declare no conflicts of interest.

## Data Availability

The data that support the findings of this study are available from the corresponding author upon reasonable request.
